# Complications of Outpatient and Inpatient Renal Biopsy: A Systematic Review and Meta-Analysis

**DOI:** 10.3390/diagnostics11040651

**Published:** 2021-04-03

**Authors:** Shih-Yi Lin, Cherry Yin-Yi Chang, Cheng-Chieh Lin, Wu-Huei Hsu, I.-Wen Liu, Chia-Der Lin, Chia-Hung Kao

**Affiliations:** 1 Graduate Institute of Biomedical Sciences and School of Medicine, College of Medicine, China Medical University, Taichung 404, Taiwan; oasisbestonlylin@gmail.com (S.-Y.L.); d4754@mail.cmuh.org.tw (C.Y.-Y.C.); cclin@mail.cmuh.org.tw (C.-C.L.); Hsuwh@mail.cmuh.org.tw (W.-H.H.); d6355@mail.cmuh.org.tw (C.-D.L.); 2 Division of Nephrology and Kidney Institute, China Medical University Hospital, Taichung 404, Taiwan; 3 Department of Teaching, China Medical University Hospital, Taichung 404, Taiwan; A24597@mail.cmuh.org.tw; 4 Department of Gynecology, China Medical University Hospital, Taichung 404, Taiwan; 5 Department of Family Medicine, China Medical University Hospital, Taichung 404, Taiwan; 6 Department of Chest Medicine, China Medical University Hospital, Taichung 404, Taiwan; 7 Department of Otolaryngology, China Medical University Hospital, Taichung 404, Taiwan; 8 Department of Nuclear Medicine and PET Center, China Medical University Hospital, Taichung 404, Taiwan; 9 Department of Bioinformatics and Medical Engineering, Asia University, Taichung 404, Taiwan; 10 Center of Augmented Intelligence in Healthcare, China Medical University Hospital, Taichung 404, Taiwan

**Keywords:** outpatient, inpatient, renal biopsies, systematic review

## Abstract

**Background:** The evidence indicates that the optimal observation period following renal biopsy ranges between 6 and 8 h. This systematic review and meta-analysis explored whether differences exist in the complication rates of renal biopsies performed in outpatient and inpatient settings. **Methods:** We searched the MEDLINE, EMBASE, and the Cochrane Database of Systematic Reviews from 1985 to February 2020. Two reviewers independently selected studies evaluating the bleeding risk from renal biopsies performed in outpatient and inpatient settings and reviewed their full texts. The primary and secondary outcomes were risks of bleeding and major events (including mortality) following the procedure, respectively. Subgroup analysis was conducted according to the original study design (i.e., prospective or retrospective). Odds ratios (ORs) and 95% confidence intervals (CIs) were calculated using a random effect meta-analysis. Heterogeneity was assessed using the *I*^2^ test. **Results:** Data from all 10 eligible studies, which included a total of 1801 patients and 203 bleeding events, were included for analysis. Renal biopsies in outpatient settings were not associated with a higher bleeding risk than those in inpatient settings (OR = 0.81; 95% CI, 0.59–1.11; *I*^2^ = 0%). The risk of major events was also comparable across both groups (OR = 0.45; 95% CI, 0.16–1.29; *I*^2^ = 4%). **Conclusions:** Similar rates of bleeding and major events following renal biopsy in outpatient and inpatient settings were observed.

## 1. Introduction

Renal biopsy, a gold standard diagnostic tool in clinical nephrology, has been used for more than a century [1,2]. Renal biopsy provides detailed histopathological information for glomerular, tubulointerstitial, and vascular renal diseases that can inform prognosis and patient management [3]. Technological advances explain the emergence of percutaneous ultrasound-guided automated needle renal biopsy as a fundamental diagnostic approach for renal tissue diagnosis; nevertheless, some contraindications exist [4]. Aside from its advantages, renal biopsy also carries a risk of complications, the majority of which are bleeding (including insignificant perirenal hematoma, microhematuria, and macrohematuria). Their prevalence rate ranges between 0.8% and 5% [5,6].

Risk factors and predictors for bleeding complications of percutaneous renal biopsy include hypertension, amyloidosis, platelet counts of ≤120 × 103 µL, a blood urea nitrogen concentration of ≥60 mg/dL, and high serum creatinine [7,8,9,10]. 

Most studies have indicated that the critical time of bleeding after a renal biopsy is within 8 h of the procedure [11,12,13]. Notably, Whitter et al. recommended a postbiopsy observation period of up to 24 h; thus, inpatient management is more widely used than outpatient management [11]. With reported life-threatening complications occurring in under 0.1% of cases and most bleeding complications requiring no further intervention, renal biopsy is generally safe [11]. Studies have indicated that bleeding complication rates in outpatient and inpatient groups undergoing renal biopsy were comparable [14,15]. Although these findings are informative, to our knowledge, no evidence-based comparisons have been conducted on this issue. Whether outpatient renal biopsy carries higher bleeding risks requiring intervention than inpatient renal biopsy remains unclear and is the subject of ongoing debate.

Thus, we conducted a systematic review and meta-analysis of studies that estimated the risk of bleeding complications associated with outpatient and inpatient observation after renal biopsy.

## 2. Methods

### 2.1. Study Inclusion Criteria

#### 2.1.1. Types of Studies and Study Participants

Randomized controlled trials and case studies (both retrospective and prospective) comparing outcomes from outpatient and inpatient renal biopsy were considered for review. All study participants were considered to be eligible regardless of age, sex, underlying comorbidities, the presence of native or transplanted kidneys, or indications for renal biopsy. 

#### 2.1.2. Types of Intervention

Studies were included if they used the same procedure protocols for renal biopsy—either real-time under ultrasound guidance or performed blindly after ultrasonographic localization— in their outpatient and inpatient groups. Biopsies of the upper, middle, and lower poles of the kidney were considered for analysis. 

#### 2.1.3. Types of Outcome Measures

Bleeding complications, inclusive of hematuria, hematoma, or anemia requiring blood transfusion comprised the primary outcomes. The secondary outcomes were major events, including hypoxia, complications requiring surgical intervention, and mortality.

#### 2.1.4. Data Source

We searched the MEDLINE, EMBASE, and the Cochrane Database of Systematic Reviews from 1966 to February 2020. The terms used for searches, which were initially limited to titles and abstracts, were kidney biopsy, renal biopsy, complications, bleeding, timing, outpatient, and inpatient. A manual search for additional studies was also performed.

### 2.2. Data Extraction

Data extraction, which consisted of reviewing the abstracts and the full texts of eligible studies, was performed independently by two of the authors (SY Lin and CH Kao). In the event of disagreement regarding study eligibility, a third author (Cherry Yin-Yi Chang) was responsible for making a judgment.

### 2.3. Statistical Analysis

For categorical data, relative risks with 95% confidence intervals (CIs) of individual and pooled statistics were calculated. A random effects model applied to the included studies to represent the means of effect distributions. The *I*^2^ statistic was used to evaluate heterogeneity among the individual studies. Review Manager 5.4 software (Cochrane Reviews, London, UK) was used to perform the DerSimonian and Laird procedure for random effects meta-analysis.

## 3. Results

### 3.1. Literature Search and Characteristics of Included Studies

**Figure 1:**
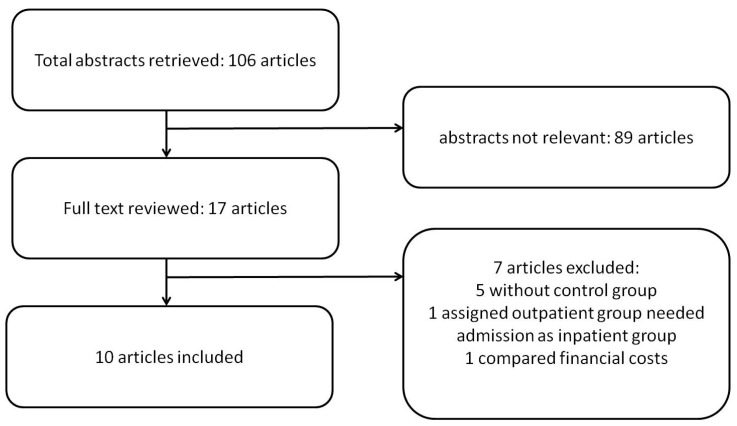
Study flow diagram.

Of the 106 articles initially retrieved, 89 articles were irrelevant. Of the 17 full articles we reviewed, seven were excluded for nonadherence to the selection criteria: five without a control group, one assigned outpatient group needed admission as an inpatient group, one compared financial cost, and another two articles were disregarded because of the unavailability of detailed information. Finally, 10 articles were included for analysis (Figure 1) [16,17,18,19,20,21,22,23,24,25]. 

**Figure 2:**
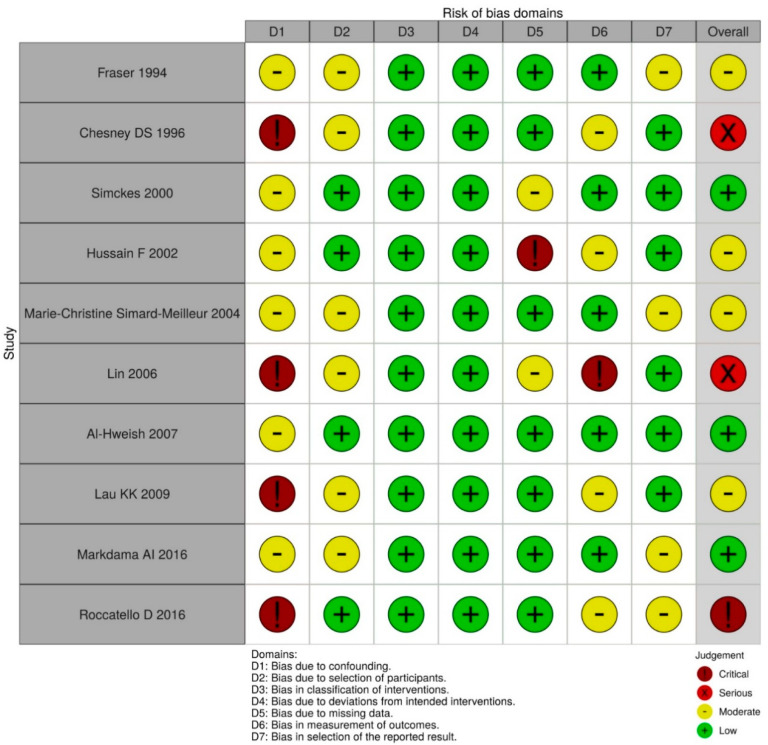
Methodological assessment.

The 10 studies included five prospective and five retrospective studies which were conducted between 1994 and 2016. Their details are presented in Table 1. All studies were conducted in populations fulfilling indications for and requiring renal biopsy. The participants in five of the studies were primarily children [17,18,21,23]. Five studies used real-time ultrasound guidance and four used ultrasound localization [19,22,23,24,25]. In four studies, biopsies were performed on both transplanted and native kidneys [18,20,21,23]. Only one of the studies in which the procedures were conducted by radiologists [25], and the procedures of other studies were performed by nephrologists. Two of the 10 studies provided final pathology reports [17,23]. The methodological quality of the included studies is summarized in Figure 2. 

### 3.2. Risk of Bleeding

**Figure 3:**
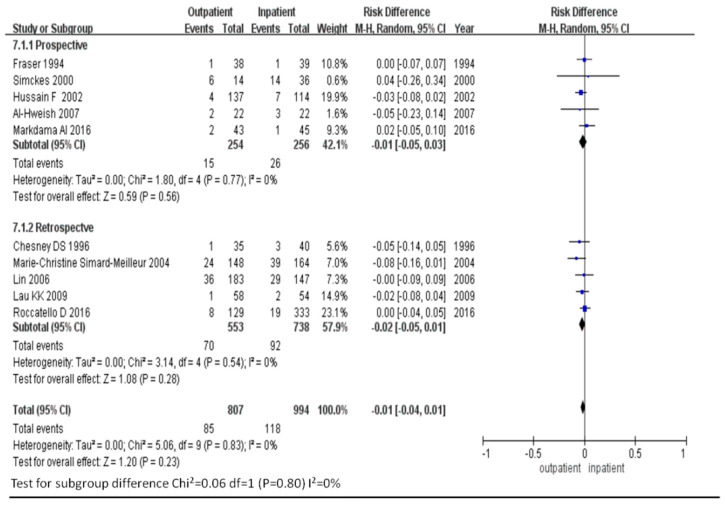
Comparison of bleeding risk in outpatient and inpatient renal biopsies.

**Figure 4:**
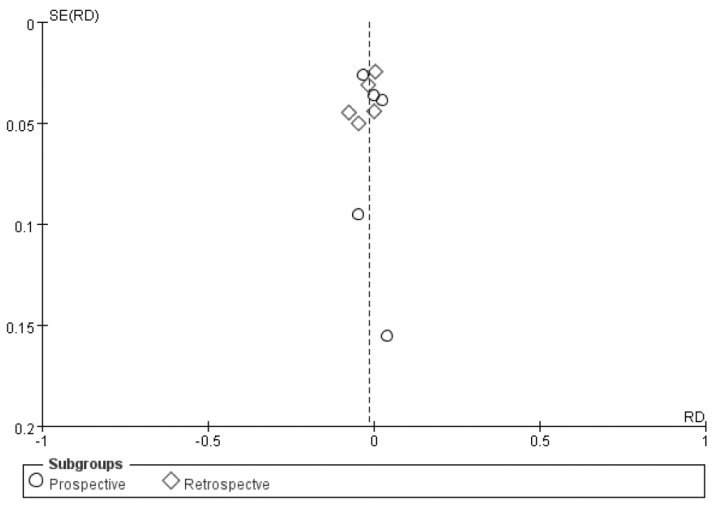
Funnel plot of effect estimates plotted against risk of bleeding.

Data from all 10 eligible studies, which included a total of 1801 patients and 203 bleeding events, were included for analysis. Bleeding risks for outpatient and inpatient renal biopsy were comparable, with an odds ratio (OR) of 0.81 (95% CI, 0.59–1.11). No heterogeneity across the studies was noted (*I*^2^ = 0; Figure 3). The funnel plot of the analysis was relatively symmetrical, indicating no publication bias (Figure 4).

**Figure 5:**
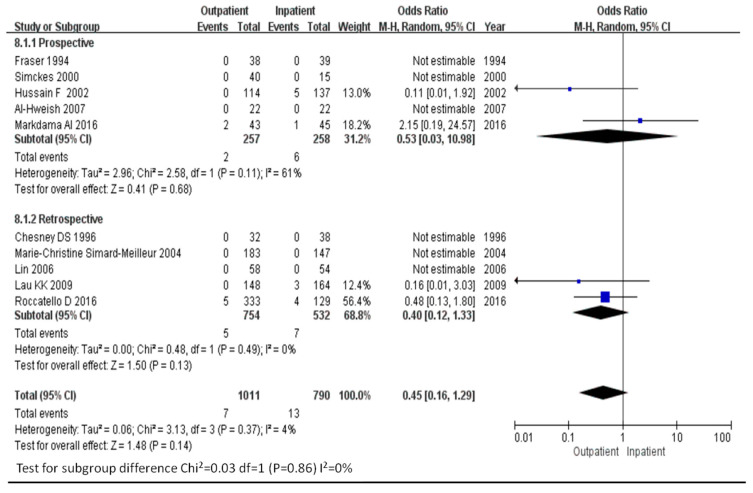
Risk of major events in outpatient renal biopsies compared with inpatient renal biopsies.

While considering types of study design, the OR of bleeding risk for outpatient renal biopsy compared with inpatient renal biopsy was 0.81 (95% CI, 0.39–1.70) in the prospective studies and 0.81 (95% CI, 0.57–1.15) in the retrospective studies, respectively. No heterogeneity across the prospective or the retrospective studies was observed (*I*^2^ = 0 for both; Figure 5).

### 3.3. Rates of Major Events

Of the 20 major events being defined as hypoxia, complications requiring surgical intervention, or mortality that occurred in all eligible studies, 7 and 13 were reported for the outpatient and inpatient groups, respectively. The risks of major events in outpatient and inpatient renal biopsy were similar (OR = 0.45, 95% CI, 0.16–1.29). The *I*^2^ statistic was 4%. 

## 4. Discussion

These meta-analysis results demonstrated that renal biopsies conducted in outpatient and inpatient settings were associated with comparable complication rates for bleeding and major events. The results have several potential implications. First, heterogeneity was present among the 10 studies considered in the meta-analysis; inpatient settings were associated with more cases of worsening renal function and bleeding tendency [16,17,18,19,20,21,22,23,24,25]. Despite the fact that the coagulation profiles of the participants in all of the included studies [16,17,18,19,20,21,22,23,24,25] were tested and that two studies reported, in detail, the participants’ underlying comorbidities [22,24], we were conservative in our analysis because of insufficient data as to whether pre-admission procedures for correcting coagulation profiles in inpatient settings were conducted. Second, the present findings can serve as a reference for financial decision making regarding the use of outpatient and inpatient management postbiopsy. As Lau et al. indicated, hospital fees for inpatient hospitalization following renal biopsy are higher than those for outpatient hospitalization [23]. Similarly, Maripuri et al. reported that inclusive of costs for intervention for any complications, renal biopsies in outpatient settings cost US$1394 per biopsy compared with US$1800 for those in inpatient settings [23]. The rise in medical care costs in recent years places a heavy burden on health care systems [25,26]. Therefore, clinicians may have an additional reason to offer the option of outpatient renal biopsy for patients not prone to bleeding. 

This meta-analysis has several limitations. First, the direct application of our conclusion to patients who underwent biopsies for transplanted kidneys allows for weaker inferences. Although four of the studies enrolled participants receiving such biopsies [18,20,21,23], studies on this specific type of renal biopsy are few. Therefore, whether graft kidney biopsies can be performed with equal safety in outpatient and inpatient settings could not be fully answered in this meta-analysis. Second, the baseline selection bias of the primary and secondary outcomes (i.e., bleeding and major events) was present in all 10 studies [16,17,18,19,20,21,22,23,24,25]. The study populations, particularly those of the retrospective studies, were not homogeneous [21,22,23,24]. Third, perirenal hematoma and arteriovenous fistula events could not be comprehensively reported because they were not systematically assessed in all studies [16,17,18,19,20,21,22,23,24]. However, because the surveillance rate of perirenal hematoma and arteriovenous fistula would be similar in outpatient and inpatient settings in each study, the ORs of their complication rates should be reasonably convincing. Fourth, only two studies presented pathology reports from the renal biopsies [17,23]; therefore, generalization of our findings to populations vulnerable to invasive procedures, such as those with antiphospholipid syndrome or ischemic heart disease, should be cautious. Furthermore, close observation of these populations during and after procedures such as renal biopsies is necessary. 

## 5. Conclusions

Our review demonstrates that the bleeding risk of outpatient renal biopsy settings were comparable with the bleeding of inpatient renal biopsy settings. Nevertheless, further high-quality evidence is warranted to confirm our findings. 

## Figures and Tables

**Table 1:** Characteristic of studies.

Study (Country)	Year	No of Patients (% Male)	Age (yrs)	Intervention Device	Biopsy Methods	Bleeding Complications
**Prospective**						
Fraser, et al. (Australia)	1994	Outpatient: 118 (43.2%)	38	14-gauge needle or Tru-Cut needle	N/A	1/38
		Inpatient: 232 (28.4%)	39			1/39
Simckes et al. (USA)	2000	Outpatient: 40 (62.5%)	12.4 ± 4.2	14- or 15-gauge needle	US pre-localization *	39%
		Inpatient: 15 (46.7%)	11.7 ± 4.0			43%
Hussain F (UK)	2002	Outpatient: 114 (NA)	12.4	16-gauge needle	US pre-localization *	4/114
		Inpatient: 137 (NA)	9.95			7/137
Al-Hweish et al. (Saudi)	2007	Outpatient: 22 (NA)	NA	16 gauge;	Real-time US	9.1%
		Inpatient: 22 (NA)	NA			13.6%
Markdama AI (Arabia)	2016	Outpatient: 43 (51%)	NA	Outpatient: 18-gauge	US pre-localization *	14%
		Inpatient: 45 (51%)	NA			8.8%
**Retrospective**						
Chesney DS (USA)	1996	Outpatient: 32 (56%)	13.4	N/A	US pre-localization * or Real time US	2/32
		Inpatient: 38 (68.4%)	11.9			6/38
Lin et al. (Taiwan)	2006	Outpatient: 183 (NA)	44.4	16-gauge needle for adult 18-gauge for pediatrics	Real-time US	19.7%
		Inpatient: 147 (NA)	50.1			19.7%
Lau KK et al. (USA)	2009	Outpatient: 58 (55%)	11.4 (1.4–20.1)	18 gauge; 10–16 cm	Real-time US	1/58
		Inpatient: 54 (53%)	13.6 (0.1–20.9)			2/54
Marie-Christine Simard-Meilleur et al. (Canada)	2014	Outpatient: 148 (47%)	53 ± 15	14, 16, or 18-gauge needle	Real-time ** US or CT	16%
		Inpatient: 164(53%)	54 ± 16			24%
Roccatello D et al. (Italy)	2016	Outpatient: 129 (49%)	52 ± 17.6	18-gauge/15 cm needle	Real-time **	6.2%
		Inpatient: 333(66%)	56 ± 19			5.7%

* US pre-localization: ultrasound pre-localization ** Real-time ultrasound: real-time ultrasound guidance; CT: computed tomography.

## Data Availability

Not applicable.

## Abbreviations

OR: Odds ratio; CI: confidence interval.
